# Reweighting of the sensory inputs for postural control in patients with cervical spondylotic myelopathy after surgery

**DOI:** 10.1186/s12984-019-0564-2

**Published:** 2019-07-25

**Authors:** Iu-Shiuan Lin, Dar-Ming Lai, Jian-Jiun Ding, Andy Chien, Chih-Hsiu Cheng, Shwu-Fen Wang, Jaw-Lin Wang, Chi-Lin Kuo, Wei-Li Hsu

**Affiliations:** 1 School and Graduate Institute of Physical Therapy, College of Medicine, National Taiwan University, Floor 3, No. 17, Xuzhou Rd., Zhongzheng District, Taipei, Taiwan; 2 Division of Physical Therapy, Department of Physical Medicine and Rehabilitation, Shin Kong Wu Ho-Su Memorial Hospital, Taipei, Taiwan; 3 Department of Surgery, National Taiwan University Hospital, Taipei, Taiwan; 4 Graduate Institute of Communication Engineering, College of Electrical Engineering and Computer Science, National Taiwan University, Taipei, Taiwan; 5 Department of Physical Therapy, Graduate Institute of Rehabilitation Science, China Medical University, Taichung, Taiwan; 6 School of Physical Therapy and Graduate Institute of Rehabilitation Science, College of Medicine, Chang Gung University, Taoyuan, Taiwan; 7 Physical Therapy Center, National Taiwan University Hospital, Taipei, Taiwan; 8 Department of Biomedical Engineering, College of Medicine and College of Engineering, National Taiwan University, Taipei, Taiwan

**Keywords:** Sensory integration, Time-frequency analysis, Gabor transform, Center of pressure

## Abstract

**Background:**

Cervical spondylotic myelopathy (CSM) is a degenerative cervical disease in which the spinal cord is compressed. Patients with CSM experience balance disturbance because of impaired proprioception. The weighting of the sensory inputs for postural control in patients with CSM is unclear. Therefore, this study investigated the weighting of sensory systems in patients with CSM.

**Method:**

Twenty-four individuals with CSM (CSM group) and 24 age-matched healthy adults (healthy control group) were analyzed in this observational study. The functional outcomes (modified Japanese Orthopaedic Association Scale [mJOA], Japanese Orthopaedic Association Cervical Myelopathy Questionnaire [JOACMEQ], Nurick scale) and static balance (eyes-open and eyes-closed conditions) were assessed for individuals with CSM before surgery, 3 and 6 months after surgery. Time-domain and time–frequency-domain variables of the center of pressure (COP) were analyzed to examine the weighting of the sensory systems.

**Results:**

In the CSM group, lower extremity function of mJOA and Nurick scale significantly improved 3 and 6 months after surgery. Before surgery, the COP mean velocity and total energy were significantly higher in the CSM group than in the control group for both vision conditions. Compared with the control group, the CSM group exhibited lower energy content in the moderate-frequency band (i.e., proprioception) and higher energy content in the low-frequency band (i.e., cerebellar, vestibular, and visual systems) under the eyes-open condition. The COP mean velocity of the CSM group significantly decreased 3 months after surgery. The energy content in the low-frequency band (i.e., visual and vestibular systems) of the CSM group was closed to that of the control group 6 months after surgery under the eyes-open condition.

**Conclusion:**

Before surgery, the patients with CSM may have had compensatory sensory weighting for postural control, with decreased weighting on proprioception and increased weighting on the other three sensory inputs. After surgery, the postural control of the patients with CSM improved, with decreased compensation for the proprioceptive system from the visual and vestibular inputs. However, the improvement remained insufficient because the patients with CSM still had lower weighting on proprioception than the healthy adults did. Therefore, patients with CSM may require balance training and posture education after surgery.

**Trial registration:**

Trial Registration number: NCT03396055

Name of the registry: ClinicalTrials.gov

Date of registration: January 10, 2018 - Retrospectively registered

Date of enrolment of the first participant to the trial: October 19, 2015

## Introduction

**Fig. 1:**
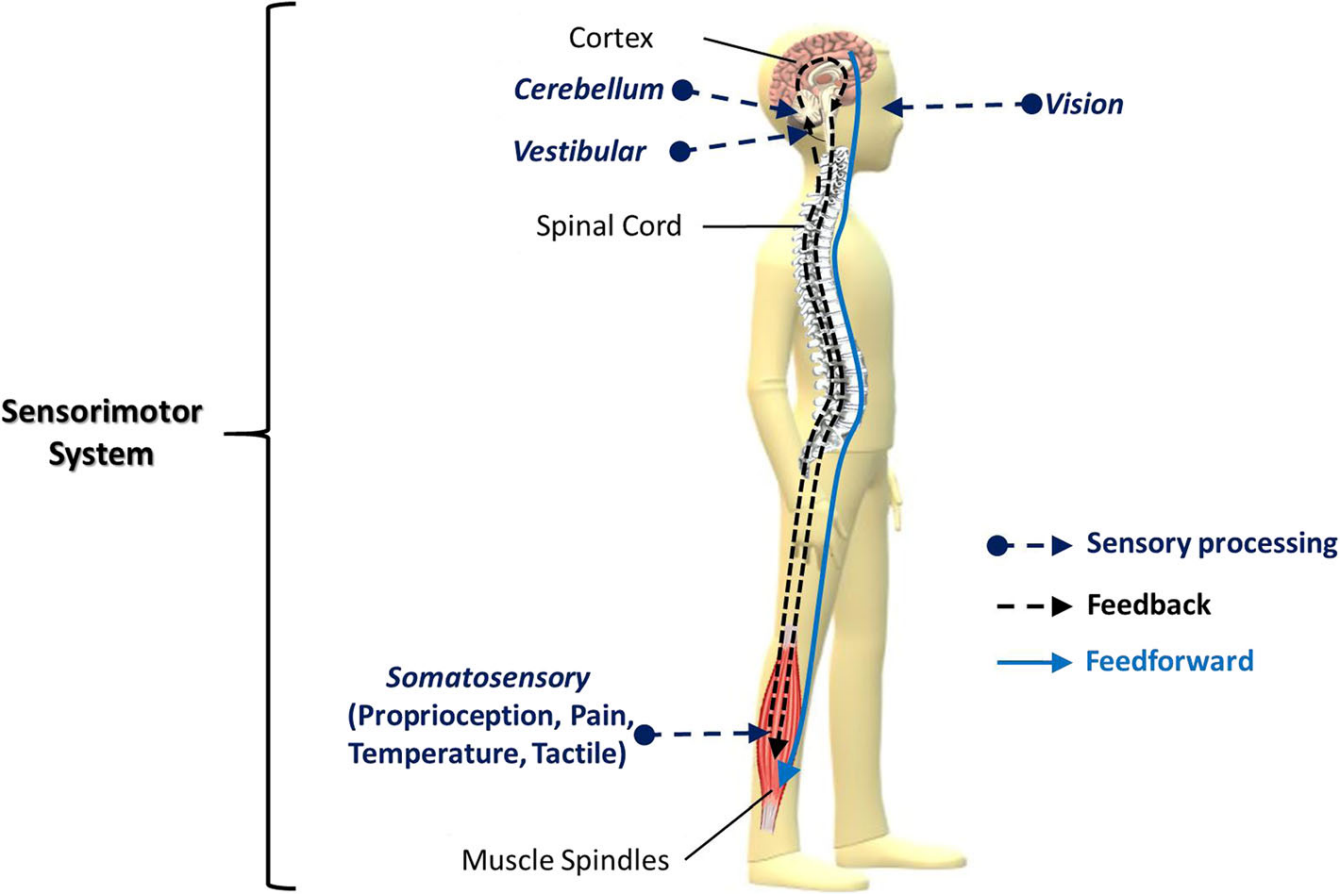
Integration of the sensorimotor system. The sensorimotor system integrates the afferent, efferent, and central nervous system, and is controlled by two control systems: feedback control and feedforward control. Feedback control involves the sensory processing, in which the cerebellar system regulates the visual, vestibular and somatosensory (i.e., proprioception, pain…etc.) inputs. The sensory feedback is conveyed to the cortex to be processed, and the reactive motor command is descended to the muscle properties. Feedforward control is described as an anticipatory action with a direct descending command without sensory feedback

Cervical spondylotic myelopathy (CSM) is a degenerative disease of the cervical spine. CSM is caused by progressive degeneration of the cervical spine and changes to the surrounding tissues [1]. Adjacent tissues and abnormal cervical motions may narrow the cervical canal and compress the spinal cord. The compression of the spinal cord can cause various neurological symptoms, such as paresthesia, muscle weakness, muscle spasm, and gait disturbance [2]. Moreover, patients with CSM exhibited postural instability compared with healthy adults [3]. Postural instability may result from impairment in proprioception and the integration of the somatosensory system, which is controlled by the neural tract in the spinal cord [3, 4]. Postural control is controlled by the sensorimotor system, which is associated with the visual, vestibular, cerebellar, and proprioceptive systems [5] (Fig. 1). When any sensory system is impaired, other sensory systems may over-activate to compensate for the insufficiency of the impaired sensory system [6]. However, understanding regarding the compensatory sensory weighting in the postural control of patients with CSM is limited.

Spinal decompression surgery is a recommended treatment aimed to remove the compressed structure and enlarge the space of spinal canal [7]. Surgical decision-making is critical for clinicians and patients. Moreover, various symptoms and signs may affect the prognosis of the surgery. Previous studies have provided inconsistent results regarding the effectiveness of surgery on the gait performance of patients with CSM [8, 9]. Furthermore, no study has discussed postural control in patients with CSM after surgery. After surgery, the proprioception in patients with CSM may recover [10]. Therefore, the sensory system may be reweighted because the demand of compensation is reduced. However, the sensory-reweighting mechanism in patients with CSM after surgery remains unknown.

To evaluate postural steadiness quantitatively, the center of pressure (COP) is often used and measured by a force platform [11]. COP displacement reflects how the body moves to maintain balance stability, and COP can be used for evaluation of the balance performance in patients with spine disorder [12]. Time-domain measures of the COP, such as mean distance and velocity, are the common methods for evaluating the postural steadiness. However, time-domain measures reflect only the final integration of sensorimotor systems without indicating the underlying mechanism. Furthermore, balance performance may not be reflected by time-domain measures when people use various control strategies, such as a rigid pattern which might be associated with the fear of falling [13]. Therefore, a new approach is required to analyze the COP signal for understanding the integration of sensorimotor systems in postural control.

Time–frequency analysis of COP was suggested for investigating the postural control of the human body [14]. The wavelet transform, which is a type of time–frequency analysis, was applied to describe the characteristics of body sway and displacement [14]. The input COP displacements were considered linear combination of several wavelets [14]. In a previous study, the time–frequency approach was used to investigate the impact of age and injuries on the balance mechanism [15, 16]. However, no study has applied the time–frequency analysis to investigate the changes in the postural control mechanism of patients with CSM after surgery.

**Fig. 2:**
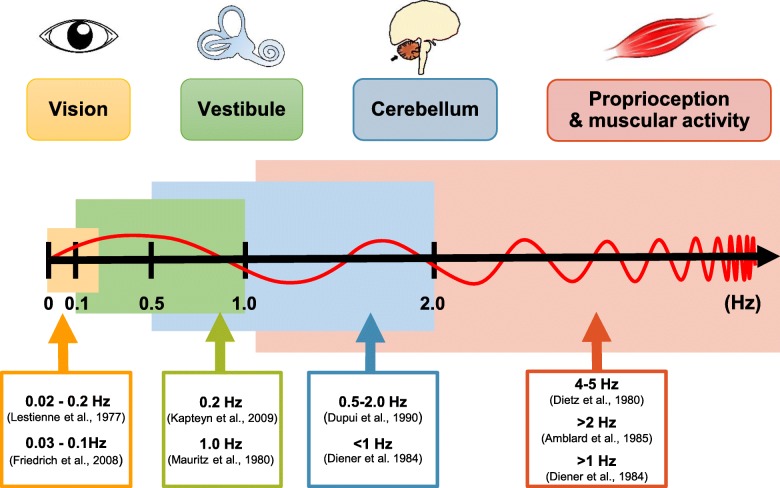
Frequency band of the sensory systems

In time-frequency analysis, each frequency band is considered an afferent of each subcomponent of the sensory system [17]. The correspondence of sensory systems and frequency bands is illustrated in Fig. 2 [18–26]. Since the frequency band of each sensory system may be overlapped, the classifications of frequency band were various in the previous studies. In the early studies of time–frequency analysis, the authors classified the sensory system into two or three frequency bands [27, 28]. In recent studies, authors have classified the sensory system into four frequency bands [29–31]. Therefore, we analyzed the COP data in four distinct frequency band: moderate-frequency (1.56 to 6.25 Hz), low-frequency (0.39 to 1.56 Hz), very-low-frequency (0.10 to 0.39 Hz), and ultralow-frequency (less than 0.10 Hz) [29–31]. This new approach may provide additional information regarding the postural control in patients with CSM during quiet standing. It may also be used as an assessment tool by clinicians for surgery decision-making.

As indicated by the aforementioned literature review, the sensory weighting mechanism in patients with CSM is unclear. Therefore, the purpose of this study was to investigate the difference of sensory weighting in the postural control of patients with CSM and healthy adults. We hypothesized that the weighting of the sensory systems in the postural control of patients with CSM may be different from that in healthy adults. We also hypothesized that the sensory systems in postural control would be reweighted after surgery.

## Material and methods

**Fig. 3:**
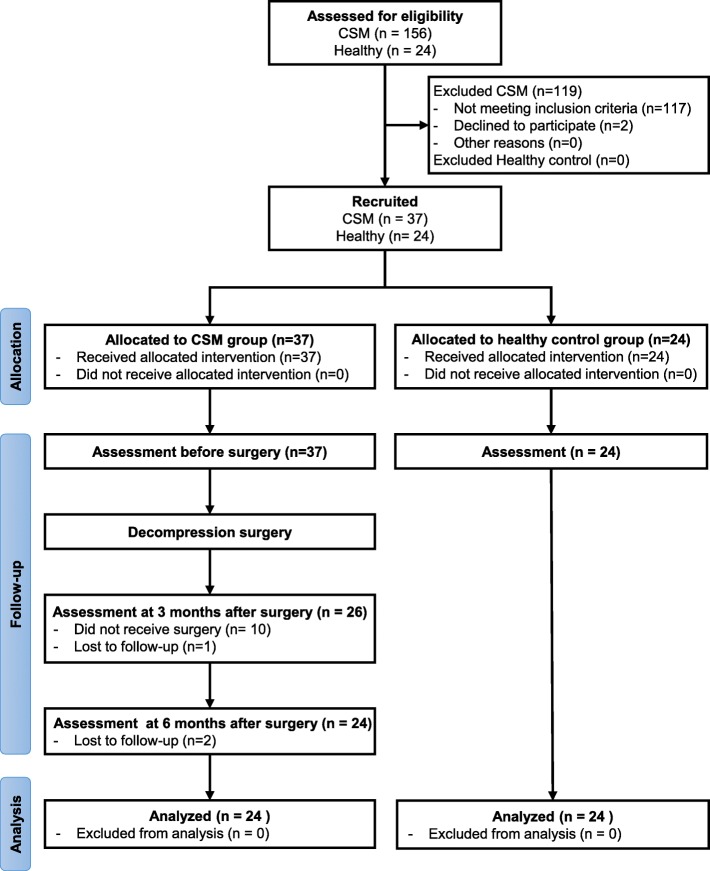
Flow diagram of this study

This paper describes a prospective longitudinal study with 6-months follow-up observing the balance performance and functional outcome in individuals with CSM after surgery. The Institutional Review Board of National Taiwan University Hospital approved this study according to the Declaration of Helsinki. The study was registered at ClinicalTrial.gov (NCT03396055). The flow diagram of this study is illustrated in Fig. 3. The experiment procedure was explained to the participants at the Department of Surgery in the National Taiwan University Hospital.

### Participants

The participants were divided into a CSM group and a healthy control group. Patients included in the CSM group were: 1) aged between 40 and 80 years; 2) with a confirmed diagnosis of CSM according to relevant image findings and neurological symptoms by surgeon; 3) with a Nurick scale at least 2; 4) able to stand for at least 30 s. Individuals with traumatic spinal injury or spinal infection, pathological musculoskeletal diseases, other neurological diseases, or orthopedic problems that may influence the ambulation were excluded. The inclusion criterion for the control group was healthy adults aged between 40 and 64 years. Participants with a history of severe or acute musculoskeletal injury to the lower extremities or spine, vestibular dysfunction, neurological dysfunction, neurosurgery, or, neck pain were excluded.

### Data collection

Data were collected from the neurosurgery clinic of National Taiwan University Hospital. The demographic, anthropometric, and standing balance assessment data were collected for all the participants. Functional outcomes were evaluated in the CSM group by the lower extremity function of modified Japanese Orthopaedic Association Scale (mJOA-LEF) [32], the lower extremity function of Japanese Orthopaedic Association Cervical Myelopathy Evaluation Questionnaire (Chinese version; JOACMEQ-LEF) [33–35], and the Nurick scale [36]. The mJOA-LEF and JOACMEQ-LEF were assessed to evaluate the functional performance, and the Nurick scale was assessed to evaluate the walking ability. All the functional outcomes of the CSM group were evaluated at three time points: before surgery (baseline), 3 months after surgery, and 6 months after surgery.

The center of pressure (COP) was recorded for the CSM group at three time points and once for the control group. All the participants were instructed to stand barefoot for 35 s on a force platform (Kistler 9286A, Kistler Instrument AG, Winterthur, Switzerland), but only the middle 30 s of 35-s test period were analyzed [11, 37]. Participants were asked to stand in a natural stance (feet shoulder-width apart) under the eyes-open (EO) and eyes-closed (EC) conditions with their arms along the sides of their body. Two successful trials were collected for each standing condition. The foot positions were marked on a script paper to ensure that the feet were placed in the same position.

### Data analysis

Signals extracted from the force platform were converted from analog-to-digital at a sampling rate of 1000 Hz. LabVIEW software (National Instruments Corp., Austin, TX, USA) was used to calculate the COP from the ground reaction force and moment in the anterior-posterior (AP) and medial-lateral (ML) directions. The 30 s of COP data were analyzed in the time domain and time–frequency domain by MATLAB (The Mathworks Inc., Natick, MA, USA). In this study, only the AP direction is reported because the previous study has shown that the postural sway in AP direction was affected in patients with CSM [38].

For time-domain analysis, the COP signal was filtered through a fourth-order zero phase Butterworth low-pass digital filter at 5 Hz. The COP mean velocity, which was defined as the average velocity of COP in the AP direction, was calculated using the following equation (Eq. (1)).

1 $$ COP\  mean\ velocity=\frac{1}{T}\sum \limits_{n=1}^{N-1}\left| AP\left[n+1\right]- AP\left[n\right]\right| $$

In aforementioned equation, AP[*n*] is defined as the COP position relative to the mean COP in the AP direction. *N* is the number of analyzed data points, and n is the number of given data points. For time–frequency-domain analysis, the COP signal was transformed using the Gabor transform, which is a time–frequency analysis method used to extract the time-variant feature of the signals [39]. The Gabor transform appears in Eq. (2).

2 $$ G\left(t,f\right)={\int}_{-\infty}^{\infty }x\left(\tau \right){\mathrm{e}}^{-\sigma \pi {\left(t-\tau \right)}^2}{\mathrm{e}}^{-j2\pi f\tau} d\tau $$

The Gabor transform uses the Gaussian function as a window to extract the local part of the COP signal (*x*(*t*)) before performing the Fourier transform [39]. In Eq. (2), the parameter *σ* controls the width of the Gaussian window. The output *G*(*t*, *f*) can indicate the COP location at different frequencies. The integral of *G*(*t*, *f*) along the *f*-axis indicates the total amount of information coming from different frequency bands. The integral is termed as the local energy content, which does not have a unit, according to Ohm’s law [40].

Moreover, the local energy content of the COP signal is divided into four distinct frequency bands, namely the (1) moderate-frequency band (1.56–6.25 Hz), (2) low-frequency band (0.39–1.56 Hz), (3) very-low-frequency band (0.10–0.39 Hz), and (4) ultralow-frequency band (below 0.1 Hz), which represent the spinal reflexive loop and muscle activity of the lower limbs, cerebellar system, vestibular system, and visual system, respectively. The local energy content of each frequency band was calculated and represented in terms of the percentage of total energy [31, 41].

### Statistics

Statistical analysis was performed using PASW Statistics 18 for Windows (SPSS, Chicago, IL, USA). The normality of the data was determined using the Shapiro–Wilk test. An independent *t* test was performed to compare the age, height, and weight of the two groups, and chi-square testing was performed to compare sex. None of COP variables were normally distributed. Therefore, the within-group comparisons of all variables were tested using the Friedman test at a statistical significance level (α) of 0.05. Multiple comparisons between each time point were tested through the Wilcoxon signed-rank test with Bonferroni correction (α/3), at a significant level of 0.016. Between-group comparisons of all the variables were performed using the Mann–Whitney U test at a significant level of 0.05.

## Results

In this study, 61 adults were recruited from National Taiwan University Hospital, and 48 adults were consequently analyzed (CSM group: *n* = 24, healthy control group: *n* = 24). The demographics and functional outcomes of the participants are described in Table 1. No significant difference was observed in the sex, age, height, or weight of the CSM group and control groups.

**Table 1:** Characteristics for all participants

	CSM group *n* = 24	Healthy control group *n* = 24	*p*-value
Sex (n, male/female)	20/4	14/10	0.06
Age (years)	59.1 ± 10.0	57.6 ± 8.0	0.60
Height (cm)	165.9 ± 8.3	163.9 ± 7.2	0.38
Weight (kg)	71.5 ± 15.2	67.0 ± 14.2	0.30
BMI (kg/m^2^)	25.8 ± 4.0	24.8 ± 3.9	0.37
Symptoms duration (months)	13.6 ± 14.9	–	–
Surgical method (anterior /posterior)	17/7	–	–
Had received physical therapy between 3 and 6 months after surgery? (Yes/ No)	4/20	–	–
mJOA-LEF (0–7)	5.2 ± 1.1	–	–
Nurick scale (0–5)	2.3 ± 0.6	–	–
JOACMEQ-LEF (%)	76.5 ± 27.4	–	–

*BMI* Body mass index, *mJOA-LEF* Lower extremity function of modified Japanese Orthopaedic Association Scale, *JOACMEQ-LEF* Lower extremity function of Japanese Orthopaedic Association Cervical Myelopathy Evaluation Questionnaire

### Functional outcomes

The results of functional outcomes from the mJOA-LEF, JOACMEQ-LEF, and Nurick scale are presented in Table 2. The mJOA-LEF and Nurick scale exhibited significant improvement. The mJOA-LEF significantly increased from the baseline value 3 months (*p* < 0.001) and 6 months (*p* < 0.001) after surgery. The Nurick scale significantly decreased from the baseline value 3 months (*p* = 0.01) and 6 months (*p* < 0.01) after surgery. The JOACMEQ-LEF score increased after surgery; however, it did not reach statistical significance.

**Table 2:** Scores of functional outcomes

	Baseline (mean ± SD)	3 months after surgery (mean ± SD)	6 months after surgery (mean ± SD)	*p*-value
mJOA-LEF (0–7)	5.17 ± 1.09	6.08 ± 1.06 *	6.25 ± 1.19 *	< 0.001
Nurick scale (0–5)	2.29 ± 0.55	1.17 ± 8.17 *	0.92 ± 1.01 *	< 0.001
JOACMEQ-LEF (%)	76.52 ± 27.39	81.06 ± 18.26	81.82 ± 21.61	0.94

*mJOA-LEF* Lower extremity function of modified Japanese Orthopaedic Association Scale, *JOACMEQ-LEF* Lower extremity function of Japanese Orthopaedic Association Cervical, Myelopathy Evaluation Questionnaire, *SD* standard deviation

*Indicated significant difference compared to the baseline: *p* < 0.05/3 = 0.016

### Standing balance assessment

#### Time domain

**Fig. 4:**
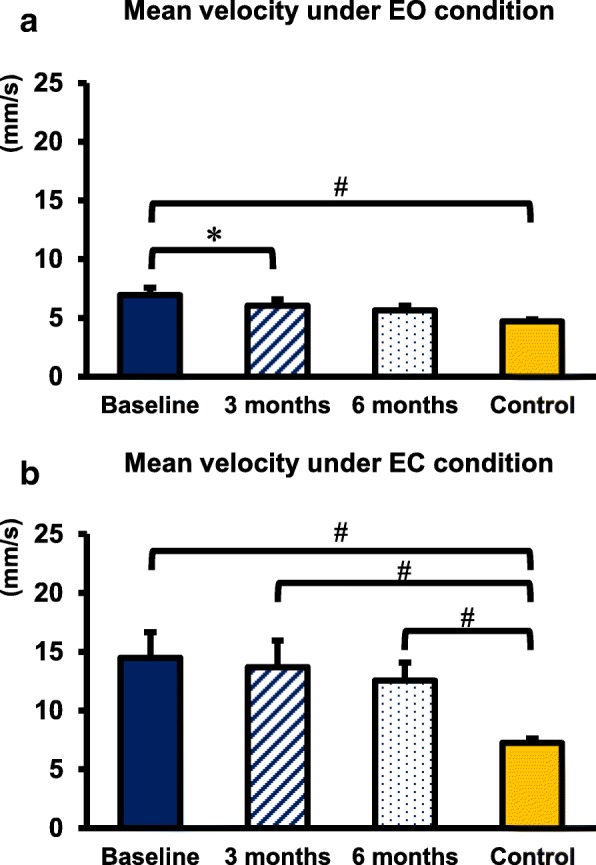
COP mean velocity at 3 time points in the CSM group and the control group. **a** COP mean velocity under EO condition. **b** COP mean velocity under EC condition. # Indicated a significant difference between the CSM group and the control group: *p* < 0.05. * Indicated a significant difference within the CSM group before and after surgery: *p* < 0.05/3 = 0.016

Under the EO condition, the COP mean velocity significantly decreased 3 months after surgery (*p* = 0.015) within the CSM group. The COP mean velocity of the CSM group was significantly higher than that of the control group at baseline (*p* < 0.01; Fig. 4a).

Under the EC condition, no significant difference was observed in COP mean velocity after surgery and at baseline within the CSM group. The COP mean velocity was significantly higher in the CSM group than in the control group at all time points (baseline: *p* < 0.001, 3 months: *p* = 0.01, 6 months: *p* < 0.01; Fig. 4b).

#### Time–frequency domain

**Fig. 5:**
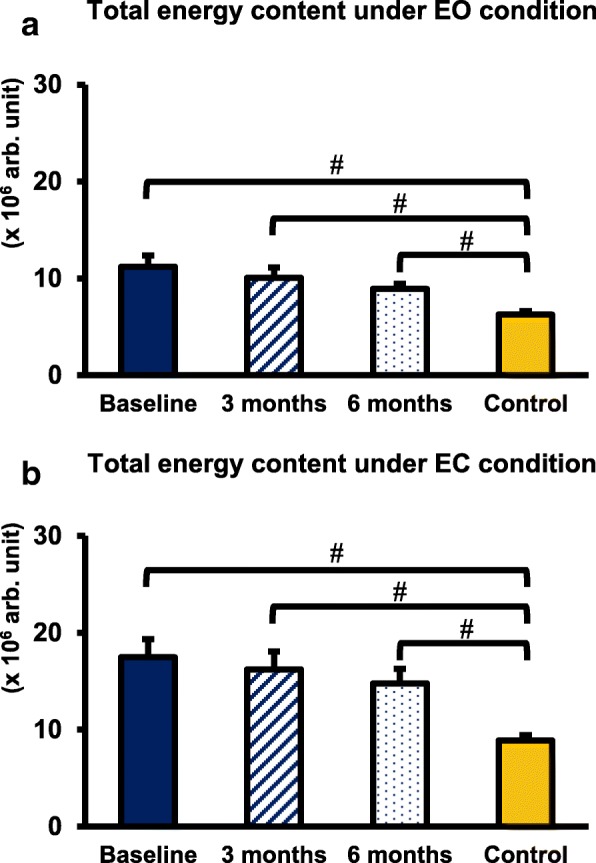
Total energy content at 3 time points in the CSM group and the control group. **a** Total energy content under EO condition. **b** Total energy content under EC condition. # Indicated a significant difference between the CSM group and the control group: *p* < 0.05

In all conditions, the total energy content decreased after surgery. However, the decrease was not statistically significant in the CSM group. At all time points, the total energy content in the CSM group was higher than that in the control group under the EO (baseline: *p* < 0.001, 3 months: *p* < 0.01, 6 months: *p* < 0.001) and EC (baseline: *p* < 0.001, 3 months: *p* < 0.01, 6 months: *p* < 0.001) conditions (Fig. 5).

**Fig. 6:**
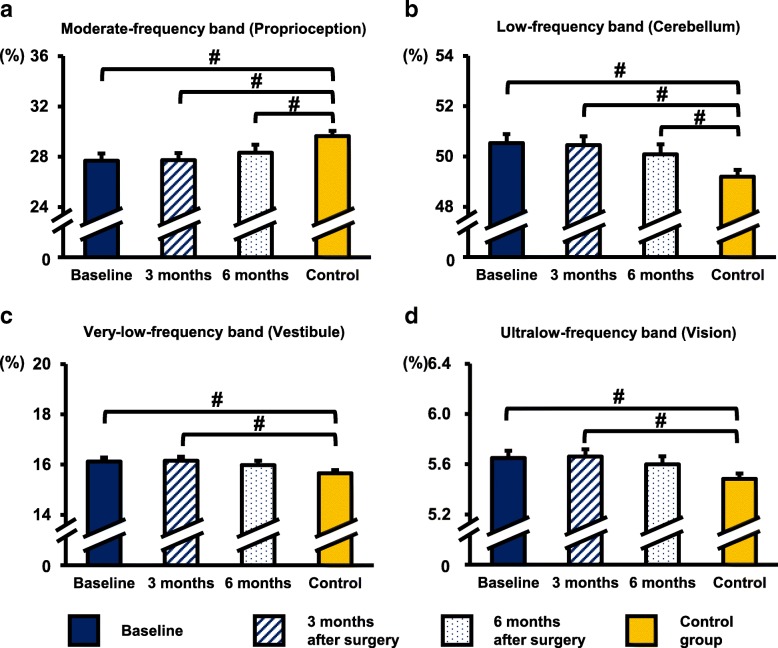
Percentage of energy content in each frequency band under the EO condition. **a** Moderate-frequency band (1.56–6.25 Hz, proprioception and spinal reflexive loop). **b** Low-frequency band (0.39–1.56 Hz, cerebellar system). **c** Very-low-frequency band (0.1–0.39 Hz, vestibular system). **d** Ultralow-frequency band (< 0.1 Hz, visual system). # Indicated a significant difference between the CSM group and the control group: *p* < 0.05

Under the EO condition, no significant difference was observed in the percentage of the four frequency bands after surgery within the CSM group. At all times points, the moderate-frequency band was significantly lower in the CSM group than in the control group (baseline: *p* < 0.01, 3 months: *p* < 0.01, 6 months: *p* = 0.04). The low-frequency band had significantly higher energy content in the CSM group than in the control group (baseline: *p* < 0.01, 3 months: *p* < 0.01, 6 months: *p* = 0.03). The very-low- and ultralow-frequency band had significantly higher energy in the CSM group than in the control group at baseline (very-low: *p* < 0.01, ultralow: *p* < 0.01) and 3 months after surgery (very-low: *p* < 0.01, ultralow: *p* < 0.01 Fig. 6).

**Fig. 7:**
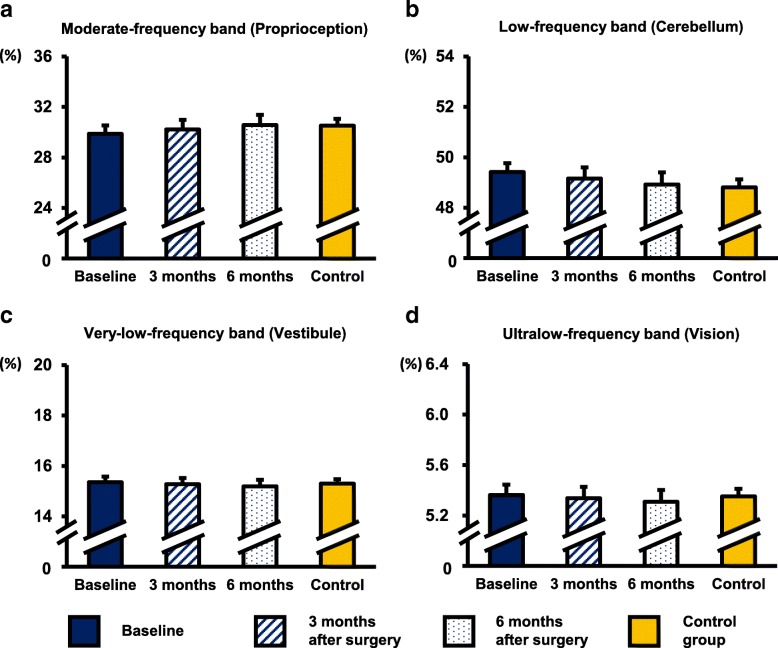
Percentage of energy content in each frequency band under the EC condition. **a** Moderate-frequency band (1.56–6.25 Hz, proprioception and spinal reflexive loop). **b** Low-frequency band (0.39–1.56 Hz, cerebellar system). **c** Very-low-frequency band (0.1–0.39 Hz, vestibular system). **d** Ultralow-frequency band (< 0.1 Hz, visual system)

Under the EC condition, no significant difference was observed in the frequency bands after surgery within the CSM group. No significant difference were observed in the four frequency bands of the two groups at any time points (Fig. 7).

## Discussion

This study investigated the difference of sensory weighting in the postural control of healthy adults and patients with CSM after surgery. The results of this study supported our hypotheses that the postural control of patients with CSM is less efficient than that of healthy adults before surgery. The low efficiency in the postural control of the patients with CSM was caused by insufficient proprioceptive inputs, as indicated by the decreased percentage of the moderate-frequency band. After surgery, postural control improved marginally, with less compensation from the visual and vestibular inputs compared with the baseline values. However, the patients with CSM still exhibited inefficient postural control.

After surgery, the functional outcome indicated by the mJOA-LEF and Nurick scale improved; however, the functional outcome indicated by the JOACMEQ-LEF score did not. The mJOA results were consistent with the results of previous studies, in which the mJOA and Nurick scale have been demonstrated to improve 3 and 6 months after surgery in patients with CSM [42, 43]. Moreover, a previous study that used the functional independence measure for assessing locomotion in patients with CSM reported a positive surgical effect on locomotion [44]. In this study, the mJOA and Nurick scale provided evidence of the positive effect of surgery on walking ability.

To consider the effect of post-surgical rehabilitation, 4 patients with CSM in this study reported that they had treated with physical therapy between 3 months and 6 months after surgery. Only one of these 4 patients tended to improve between 3 months and 6 months after surgery. The types of physical therapy they received after surgery included cervical range of motion exercise, cervical traction and modalities intervention. These interventions may improve the range of motion of cervical joint, but may not directly affect the balance performance of patients with CSM. Therefore, the improvement of balance performance was mostly contributed from the surgery in this study.

In this study, the patients with CSM exhibited insufficient muscular activity and a spinal reflexive loop from the lower extremities. The patients with CSM required the visual, vestibular, and cerebellar systems to be over-activated to compensate for the aforementioned insufficiency. Similar results with large COP displacements have been obtained for patients with CSM [38, 45]. According to our results, the proprioception input decreased with compensation from other sensory systems; this is in agreement with previous research on postural control in patients with neck pain [31]. The decreased recruitment of the muscular proprioceptive system was countered by the increasing usage of the vestibular system in patients with neck pain [31].

The unsteady balance performance may be caused by the blockage of information transmission along the lateral corticospinal tract and dorsal spinocerebellar tracts, which are responsible for movement and proprioceptive feedback, respectively. Poor joint proprioception leads to poor estimation of body position [4], and a prolonged conduction time of the corticospinal tract may cause delayed body adjustment [46].

Under the EC condition, the patients with CSM required increased sensorimotor inputs to cope with the postural unsteadiness, as indicated by the increased total energy content. The aforementioned result is consistent with a study that investigated the influence of vision on postural control [41]. Although our study indicated the increased demand of sensory feedback in patients with CSM, we did not observe differences between the two groups in the weighting of each sensory system. The possible reason for this observation is that standing with EC condition may be too challenging for CSM patients, as indicated by the increased weighting on proprioceptive system.

Under the EO condition, the postural sway decreased 3 months after surgery in patients with CSM. Moreover, a marginal increase in the weighting of proprioceptive systems and a decrease in the weighting of visual and vestibular systems were observed in the patients with CSM after surgery. The discrepancy of compensation from the visual and vestibular systems between the patients and healthy adults diminished 6 months after surgery. This result may be an indirect evidence of the improvement in the proprioception of the lower extremities [10]. This study is the first to investigate the postural control in patients with CSM after surgery.

Under the EC condition, the postural sway of the patients with CSM decreased after surgery; however, the sway remained greater than that of the healthy adults. In a previous study, impairment in the lower extremity function commonly persisted after surgery [47], which may affect postural control in patients with CSM. Long-term compression of the spinal cord may induce cell apoptosis of the nerves in the spinal cord, causing an irreversible neurological deficit and slow recovery of the spinal cord [48]. Furthermore, metabolic changes in the cortex may decrease the recovery in motor and sensory functions for patients with CSM [49]. Therefore, the postoperative training must be used for providing new stimulation to the neurons of the spinal cord and enhancing cortical plasticity.

In addition to the neural tract, proprioception from the musculoskeletal system may be altered because of degenerative changes or the surgical realignment of the cervical spine. Effective postural control requires coordination among multiple joints [50, 51]. Structural changes in joint position and alignment may affect joint coordination when controlling posture for adaptation [41, 52]. In a previous study has shown that an adaptive strategy of postural control was developed for maintaining balance in patients with spinal stenosis after surgery [52]. Therefore, changes in cervical alignment caused by internal factors, such as degeneration, or external factors, such as surgery, may contribute to proprioceptive insufficiencies around the neck [30]. Furthermore, changes in cervical alignment may affect head position and body orientation, resulting in poor balance [53, 54].

Time–frequency analysis has been used to analyze the COP signal for postural control in pathological diseases [29, 30, 41]. Our team was the first to use Gabor transform wavelet analysis for analyzing the COP time–frequency domain [39]. In contrast to discrete wavelet analysis, which has been performed in many previous studies, Gabor transform wavelet analysis can be used to decompose the COP signal and obtain the instantaneous frequency and local characteristics of the original signal with a fluctuating frequency [39]. The Gabor transform can effectively extract noise from the signal. Therefore, the energy content obtained with the Gabor transform is an accurate approximation of the real energy of the signal. Furthermore, the energy content of the COP signal in each frequency band can be represented by a spectrogram with warm colors for high energy and cold colors for low energy [14]. The color mapping of each frequency band may be used as bio-feedback for balance training in future studies.

Discrete wavelet analysis has been used in patients with adolescent idiopathic scoliosis. In discrete wavelet analysis, the energy content is decomposed into three distinct frequency bands, which represent the somatosensory, vestibular, and visual systems [41]. In this study, we decomposed the energy content into four distinct frequency bands, which represent the proprioceptive, cerebellar, vestibular, and visual systems. This quadripartite classification can provide a comprehensive understanding of the sensory system in various challenging tasks, including visual disturbance and multitasking.

The energy content of the moderate-frequency band of this study was higher than observed by Sim et al. [41], whereas the energy content of the ultralow-frequency band of this study was lower than that observed by Sim et al. These discrepancies may arise from the age difference of the participants in the two studies. The participants in our study were older adults; however, the participants in the study of Sim et al. were adolescents. In older adults, increased muscle activity may compensate for insufficient visual feedback [55]. This compensation strategy may have caused the increased energy content in the moderate-frequency band and decreased percentage of the ultralow-frequency band of our study. The difference may also result from the different methods of time–frequency analysis used. The Gabor transform can accurately provide the energy content of each frequency band with specific cutoff points, whereas discrete wavelet analysis can decompose the signal of each frequency band only with approximate cutoff points.

In this study, the underlying mechanism of postural control in patients with CSM was investigated using a modern mathematical method. This method can simultaneously consider the time and frequency domains in the signal process. Moreover, the effect of surgery on the postural control in patients with CSM was investigated after 6 months. The compensatory changes in the neurological system of the patients with CSM were also observed after surgery. Our study indicated that simple stabilometry can be used in clinical settings for assessing the sensory weighting in the postural control of patient populations. In future studies, a large sample size can be used, and patients can be divided into subgroups based on the severity of CSM. Postural assessment can be used as a diagnostic tool to assist in surgical decision-making. Furthermore, the compensatory strategy existed even after surgery, which implies that postoperative balance training is required for patients with CSM to prevent falling and secondary injury. In future studies, simulations of falling and task-specific training, such as perturbation-based training, can be applied to patients with CSM to accelerate the reweighting of postural control systems and stimulate neuromuscular modulation [56, 57].

Our study has several limitations. First, static balance was not preaccessed to identify the eligible patients. The Nurick scale was used in this study to select potential patients with balance disturbance. However, the Nurick scale addresses ambulation problems rather than balance disturbance [36]. Therefore, the participants included in this study may have had minor balance issues. Second, the patients had disparate symptoms and disease durations, which caused a large standard deviation in the CSM group. The onset of CSM symptoms was unclear or vaguely reported by the patients in a subjective manner. Recruiting patients with a similar duration and severity of CSM was difficult. However, we performed the first assessment within 1 month before surgery to ensure that the baseline was consistent for all the patients. Lastly, we did not conduct long-term follow-ups 1 or 2 years after surgery. Although this study has several limitations, it provides results of a scientific investigation of the postural control and effect of surgery in patients with CSM. In future studies, a larger sample size should be used and long-term outcomes should be investigated.

## Conclusions

Before surgery, the patients with CSM may have had inefficient postural control with compensatory sensory weighting, as indicated by the decreased weighting on proprioception and increased weighting on the other three studied sensory inputs. After surgery, postural control improved, with low compensation from the visual and vestibular inputs in the patients with CSM. However, the improvement was insufficient because the weighting on proprioception was lower in the patients with CSM than in the healthy adults. The COP energy content analyzed through the Gabor wavelet transform can enable further understanding of human postural control. In future studies, long-term follow-up after surgery should be conducted. Furthermore, patients with CSM may require balance training and posture education after surgery to prevent falling and secondary cervical injury.

## Data Availability

Data are available from the authors upon reasonable request.

## Abbreviations

AP: Anterior-posterior
COP: Center of pressure
CSM: Cervical spondylotic myelopathy
EC: Eyes-closed
EO: Eyes-open
JOACMEQ-LEF: Lower extremity function of the Japanese Orthopaedic Association Cervical Myelopathy Evaluation Questionnaire
mJOA-LEF: lower extremity function of the modified Japanese Orthopaedic Association Scale
ML: Medial-lateral
